# The effect of the glucosylceramide synthase inhibitor lucerastat on cardiac repolarization: results from a thorough QT study in healthy subjects

**DOI:** 10.1186/s13023-020-01582-7

**Published:** 2020-10-27

**Authors:** Markus S. Mueller, Patricia N. Sidharta, Christine Voors-Pette, Borje Darpo, Hongqi Xue, Jasper Dingemanse

**Affiliations:** 1 Department of Clinical Pharmacology, Idorsia Pharmaceuticals Ltd, Hegenheimermattweg 91, 4123 Allschwil, Switzerland; 2 QPS Netherlands B.V., Groningen, The Netherlands; 3 eResearch Technology Inc, ERT, Rochester, NY USA

**Keywords:** Lucerastat, Glucosylceramide synthase inhibitor, Fabry disease, QT interval, Thorough QT study

## Abstract

**Background:**

Fabry disease is a rare inherited glycosphingolipid storage disorder caused by deleterious mutations in the *GLA* gene coding for the lysosomal enzyme α-galactosidase A. The glucosylceramide synthase inhibitor lucerastat is an iminosugar with potential to provide oral substrate reduction therapy in Fabry disease, regardless of the patient´s underlying mutation. Since lucerastat exhibits systemic exposure and many patients with Fabry disease suffer from rhythm and conduction abnormalities its effects on cardiac repolarization were evaluated in a thorough QT study.

**Methods:**

In Part A of this randomized, double-blind, placebo-controlled phase 1 study, single oral doses of 2000 and 4000 mg lucerastat were investigated to determine the supratherapeutic dose for Part B. The latter was a four-way crossover study to demonstrate that lucerastat at single oral therapeutic and supratherapeutic doses had no effect on the QTc interval > 10 ms using concentration-QTc modeling as primary analysis. The primary ECG endpoint was placebo-corrected change-from-baseline (ΔΔ) in Fridericia-corrected QTc (ΔΔQTcF). Open-label moxifloxacin served as positive control.

**Results:**

The effect of lucerastat on ΔΔQTcF was predicted as 0.39 ms (90% confidence interval [CI] − 0.13 to 0.90) and 1.69 ms (90% CI 0.33–3.05) at lucerastat peak plasma concentration after dosing with 1000 mg (5.2 µg/mL) and 4000 mg (24.3 µg/mL), respectively. A QTcF effect > 10 ms was excluded up to lucerastat plasma concentrations of approximately 34.0 µg/mL. Lucerastat did not exert an effect on other ECG parameters. Across doses, absorption of lucerastat was rapid, its elimination half-life ranged from 8.0 to 10.0 h, and the pharmacokinetics (PK) of lucerastat were dose-proportional. Moxifloxacin PK were in line with published data and assay sensitivity was demonstrated by the moxifloxacin QTc response. Lucerastat was safe and well tolerated.

**Conclusions:**

Lucerastat up to a dose of 4000 mg has no clinically relevant liability to prolong the QT interval or any clinically relevant effect on other ECG parameters. This will be an important factor in the overall benefit-risk assessment of lucerastat in the potential treatment of Fabry disease.

*Trial registration* The study was registered with the ClinicalTrials.gov identifier NCT03832452 (February 6th, 2019, https://clinicaltrials.gov/ct2/show/NCT03832452) and the EudraCT number 2018-004546-42 (December 17th, 2018).

## Background

Fabry disease (FD) is a rare inherited glycosphingolipid storage disorder caused by deleterious mutations in the *GLA* gene coding for the lysosomal enzyme α-galactosidase A (α-GalA). In patients with FD, the catabolic activity of α-GalA is either reduced or absent. Therefore, α-GalA substrates including globotriaosylceramide, globotriaosylsphingosine, and other neutral glycosphingolipids accumulate in lysosomes and other subcellular compartments. This manifests in progressive malfunction of many cell types and organs, particularly kidneys, heart, nervous system, and skin. Consequently, patients with FD suffer from a broad variety of clinical symptoms, including neuropathic pain, progressive renal disease, cardiomyopathy, stroke, and gastrointestinal disturbances [1, 2].

Currently, two therapeutic modalities are approved for the treatment of FD. These are enzyme replacement therapy (ERT) and pharmacological chaperone therapy. Emerging treatment strategies include substrate reduction therapy, mRNA-based therapy, and gene therapy [3–5]. ERT with bi-weekly infusion of recombinant enzyme, either agalsidase alfa or beta, aims to restore a level of α-GalA activity that is enough to clear the cytotoxic globotriaosylceramide accumulation in tissues, thereby preventing, stabilizing, or reversing the progressive decline in the function of affected organs before irreversible damage occurs [6]. Some specific *GLA* gene mutations can lead to expression of abnormal α-GalA that is not effectively delivered into the lysosome. By binding to its active site, pharmacological chaperone therapy with oral migalastat can partially restore α-GalA trafficking to the lysosomes and thereby its enzymatic activity in cells of Fabry patients with so-called ‘amenable’ mutations [7, 8].

Despite the availability of ERT and pharmacological chaperone therapy, there is still a high unmet medical need. Bi-weekly infusion of ERT can be cumbersome and/or associated with tolerability issues, and potentially induces neutralizing antibodies, jeopardizing its effectiveness [9, 10]. Pharmacological chaperone therapy with migalastat is limited to Fabry patients with specific ‘amenable’ mutations [11, 12].

Substrate reduction therapy aims to prevent accumulation of glycosphingolipids by inhibiting glucosylceramide synthase. This enzyme catalyzes the first committed step of glycosphingolipid biosynthesis and, therefore, substrate reduction therapy can reduce the rate of synthesis of globotriaosylceramide to a level compatible with its residual clearance. The desired outcome is a reduction of net globotriaosylceramide load in tissues leading to symptomatic improvement and a delayed progression towards end-stage organ failure. This therapeutic approach has been proven successful with miglustat for other lysosomal storage disorders. Miglustat is marketed under the tradename Zavesca® and indicated for the treatment of type 1 Gaucher disease and Niemann-Pick type C disease. Another substrate reduction therapy, venglustat, has been investigated in a long-term, phase 2 clinical trial (NCT02489344) to evaluate its effectiveness in male patients with FD who completed a previous phase 2 trial (NCT02228460) [4].

Lucerastat (*N*-butyldeoxygalactonojirimycin or (2R,3S,4R,5S)-1-butyl-2-(hydroxylmethyl) piperidine-3,4,5-triol) is a small iminosugar molecule that is in development to provide oral substrate reduction therapy for FD, independent of the patient´s underlying mutation. Inhibition of glucosylceramide synthase by lucerastat dose dependently lowered globotriaosylceramide and lysosomal staining in cultured fibroblasts from Fabry patients, independent of their α-GalA genotype [13]. In a phase 1b proof-of-concept study, 10 subjects with FD received oral lucerastat (1000 mg twice daily) for 12 weeks on top of ERT and 4 received ERT only [14]. Mean plasma levels of globotriaosylceramide and its precursors glucosylceramide and lactosylceramide were markedly decreased from baseline following treatment with lucerastat (after 12 weeks: − 55.0%, − 49.0%, and − 32.7%, respectively).

Following single oral doses (ranging between 100 and 1000 mg) and multiple oral doses (up to 1000 mg twice daily for up to 7 days) the PK of lucerastat in healthy male subjects were dose-proportional and characterized by quick absorption and a short half-life (t_½_) [15]. No accumulation was observed. The PK characteristics of lucerastat in subjects with FD who received 1000 mg lucerastat twice daily for 12 weeks were similar to those observed in healthy subjects [14]. In the phase 1 studies, lucerastat was safe and well tolerated, both in healthy subjects and in patients.

Currently, a double-blind, placebo-controlled phase 3 study is being conducted to determine the efficacy and safety of lucerastat oral monotherapy (1000 mg twice daily) in male and female adult subjects with a diagnosis of FD and Fabry-associated neuropathic pain, independent of their genotype (ClinicalTrials.gov identifier: NCT03425539, EudraCT number: 2017-003369-85).

Nonclinical data including long-term toxicity studies were not indicative of any QT liability of lucerastat or any other adverse effect on cardiac function. However, clinical investigation of the potential QT liability is required for all investigational drugs with systemic exposure. In addition, many patients with FD suffer from cardiomyopathies. Rhythm and conduction abnormalities are commonly seen in these patients [16, 17]. Therefore, this study was conducted to evaluate the effect of lucerastat on the QT/QTc interval, in line with applicable regulatory guidance [18, 19], and after consultation with the US Food and Drug Administration Interdisciplinary Review Team who approved both the study design and the approach to the cardiodynamic evaluation.

The primary objective of this thorough QT (TQT) study was to demonstrate that lucerastat at therapeutic and supratherapeutic doses does not exhibit an effect on the QTc interval > 10 ms based on concentration-QTc analysis as described previously [20] and recommended by regulatory guidance [18, 19].

## Methods

### Human subject protection

Both the study protocol and informed consent documents were approved by the local Ethics Committee (Medisch Ethische Toetsings Commissie, Assen, The Netherlands). Written informed consent was obtained from each subject prior to performing any study-mandated procedure. The study was registered with the ClinicalTrials.gov identifier NCT03832452 and the EudraCT number 2018-004546-42. It was conducted at QPS Netherlands B.V. (Groningen, The Netherlands) from January 28th (first screening) to April 22nd, 2019, in accordance with the revised Declaration of Helsinki principles, International Council for Harmonization Good Clinical Practice guidelines, and applicable laws and regulations. The first subject was enrolled (i.e., randomized and dosed) on February 13th, 2019.

### Study design and sample size

This was a single-center, randomized, two-part phase 1 study to assess the effect of single therapeutic and supratherapeutic doses of lucerastat on the QT/QTc interval duration. Part A was conducted as a double-blind, placebo-controlled study in healthy male subjects to determine the supratherapeutic dose of lucerastat to be used in Part B. The latter was conducted as a double-blind (for lucerastat), placebo-controlled, four-way crossover study in healthy male and female subjects including open-label moxifloxacin as a positive control to assess assay sensitivity. Both parts of the study were conducted independently of each other and subjects could only participate in one.

At Screening, subjects´ health was assessed based on medical history, previous medications, clinical laboratory tests, physical examination, vital signs, and 12-lead safety ECG. Standard criteria for clinical pharmacology studies in healthy subjects were used, including age between 18 and 55 years, body mass index (BMI) between 18 and 30 kg/m^2^, QTcF < 450 ms for male subjects and < 470 ms for female subjects, and heart rate (HR) ≤ 90 beats per minute (bpm). Fertile males and women of childbearing potential had to apply highly effective contraceptive measures.

This was an inpatient study. On days in the study center, subjects received standardized meals/snacks. On days with scheduled dosing, subjects remained fasted for at least 10 h prior to and 4 h after study treatment administration, which took place in the morning. The intake of fluids was not allowed from 1 h before until 1 h after study treatment administration except for the 240 mL of water for study treatment intake; up to 400 mL were allowed if needed to swallow all lucerastat or placebo capsules. Lucerastat was formulated as size 0 hard gelatin capsules at a dose strength of 250 mg. Placebo capsules were identical in appearance to the lucerastat capsules and contained the same inactive excipients.

In Part A, 8 male subjects were randomized to receive either lucerastat or placebo (3:1 ratio) under fasted conditions. This part was conducted because clinical experience with doses higher than the anticipated therapeutic dose of 1000 mg lucerastat twice daily was lacking. Lucerastat or placebo was administered as a single oral dose of 2000 mg on Day 1 and 4000 mg on Day 3 to investigate the safety, tolerability, and PK of both doses for the determination of the supratherapeutic dose for Part B. Subjects were confined to the clinical site from the afternoon of Day-1 until the end of study examination on Day 5.

In Part B, 36 subjects, at least 30% of each sex, were to be enrolled to achieve at least 30 evaluable subjects. Based on experience published previously [21, 22], a sample size of 30 evaluable subjects was expected to provide more than 95% power to exclude that lucerastat causes a QTc effect > 10 ms at clinically relevant plasma levels as shown by the upper bound of the two-sided 90% CI of the predicted effect on ΔΔQTcF at the observed lucerastat geometric mean maximum plasma concentration (C_max_). The calculation was based on an assumed underlying effect of lucerastat of 3 ms and a standard deviation (SD) of change-from-baseline QTcF (∆QTcF) of 8 ms. In 4 separate periods, subjects received in a random sequence (3 4 × 4 Williams squares) [23], under fasted conditions, single oral doses of the following study treatments: 1000 mg lucerastat (anticipated therapeutic dose), 4000 mg lucerastat (supratherapeutic dose), 400 mg open-label moxifloxacin (Avelox®), and placebo. Each dosing was followed by a 36-h observation period. The 4 periods were separated by a washout period of 8 days and an end of study examination took place 2–3 days after the last dosing. In each period, subjects were confined to the clinical site from the afternoon of the day before dosing until the end of the observation period on Day 2.

### Sample collection and bioanalysis

For the determination of lucerastat and moxifloxacin plasma concentrations, about 4 mL of blood was collected for each analyte pre-dose (0 h) and 0.5, 1, 1.5, 2, 2.5, 3, 3.5, 4, 6, 8, 12, 16, 24, and 36 h post-dose by direct venipuncture or via an intravenous catheter placed in an antecubital vein. In Part A, an additional blood sample was taken 48 h post-dose following administration of 4000 mg lucerastat. For bioanalytical measurement of lucerastat, blood samples were collected into potassium ethylenediaminetetraacetic acid containing tubes, and for moxifloxacin into lithium heparin containing tubes, and cooled on ice. Within 30 min after collection, blood samples were centrifuged at 4 °C at 1500*g* for 10 min. Plasma was aliquoted into polypropylene tubes and stored at − 20 °C or below. Plasma concentrations of lucerastat were determined using a validated liquid chromatography coupled to tandem mass spectrometry assay [15]; the lower limit of quantification (LLOQ) was 50 ng/mL. For lucerastat, the inter-batch precision was ≤ 4.1% and the inter-batch accuracy was in the range from − 1.2 to 1.1%. Plasma concentrations of moxifloxacin were determined using a validated liquid chromatography method coupled to tandem mass spectrometry detection after protein precipitation; the LLOQ was 10 ng/mL. For moxifloxacin, the inter-batch precision was ≤ 7.3% and the inter-batch accuracy was in the range from − 5.1 to 4.4%.

### Pharmacokinetic assessments

The plasma PK parameters of lucerastat and moxifloxacin were derived by noncompartmental analysis (Phoenix WinNonlin version 8.0; Pharsight Corp., Mountain View, CA, USA). The measured individual plasma concentrations were used to directly obtain C_max_ and the time to reach C_max_ (t_max_). The area under the plasma concentration–time curve from zero to time t (AUC_0−t_) was calculated according to the linear trapezoidal rule, using the last measured concentration above the LLOQ. All plasma concentration values below the LLOQ were set to zero. The area under the curve from zero to infinity (AUC_0−∞_) was calculated by combining AUC_0−t_ and AUC_extra_, where AUC_extra_ represented an extrapolated value obtained by the last measured plasma concentration above LLOQ divided by the terminal elimination rate constant (λ_z_). The t_½_ was calculated as ln2/λ_z_. The assumption was made that C_max_, AUC, and t_½_ were log-normally distributed.

### ECG methodology

Cardiodynamic evaluation with ECGs extracted from continuous (Holter) recordings was performed in Part B only. The continuous recording was performed in each period, running from approximately 1 h pre-dose on Day 1 until 36 h post-dose on Day 2. ECGs were recorded using an M12R digital 12-lead Holter recorder (Global Instrumentation, Manlius, NY, USA). Data were stored on memory cards. At the ECG service provider eResearch Technology Inc. (ERT®, Rochester, NY, USA), replicate, nonoverlapping 14-s ECGs were extracted in close succession within each pre-defined extraction window related to study treatment administration on Day 1 (i.e., 0.75, 0.5, and 0.25 h pre-dose; 0.5, 1, 1.5, 2, 2.5, 3, 3.5, 4, 6, 8, 12, 24, and 36 h post-dose) using the proprietary method TQT Plus® (ERT®, Rochester, NY, USA). This enabled the extraction of a high-quality data set by identifying periods of recordings with the lowest available HR variability and noise. The average of the 3 pre-dose time points on Day 1 of each period was used as baseline. Subjects were resting in a supine position for a minimum of 15 min at each time point of ECG reading (10 min before and 5 min during ECG reading). ECG intervals were measured using the Expert Precision QT technique (formerly described as High-Precision QT analysis) [24]. ECG analysts at the central lab were blinded to subject, visit, and study treatment. The same analyst was assigned for all ECGs of a given subject using lead II as the primary analysis lead in a process overseen by an experienced cardiologist. The QT and preceding RR value for each beat was used for HR correction; QTcF was derived using Fridericia’s formula defined as $${\text{QTcF}} = {\text{QT/RR}}^{{{\raise0.5ex\hbox{$\scriptstyle 1$} \kern-0.1em/\kern-0.15em \lower0.25ex\hbox{$\scriptstyle 3$}}}}$$. The median value of each ECG parameter from the set of evaluable beats in each extracted replicate was calculated and then the mean of all available medians, at minimum 3 medians, from the nominal time point was used as the subject’s reportable value at that time point.

### Safety assessments

Safety and tolerability were evaluated based on reporting of treatment-emergent AEs (TEAEs) regardless of causality, clinical laboratory tests, vital signs, physical examinations, and 12-lead safety ECGs to detect any immediate ECG effects. AEs were coded using the Medical Dictionary for Regulatory Activities (MedDRA version 22.0). The relationship to study treatment and the intensity of TEAEs was judged by the investigator.

### Statistical analysis

The primary analysis was based on concentration-QTc modeling of the relationship between lucerastat plasma concentration and ∆QTcF with the intent to exclude an effect > 10 ms at clinically relevant plasma concentrations. This relationship was quantified using a linear mixed-effects modeling approach with ∆QTcF as dependent variable, drug plasma concentration as explanatory variate (0 for placebo), centered baseline QTcF (i.e., baseline QTcF for an individual subject minus the population mean baseline QTcF for all subjects) as an additional covariate, study treatment (active = 1 or placebo = 0) and time as categorical factors, and a random intercept and slope per subject following statistical approaches described previously [20]. Additional exploratory analyses via graphical displays and/or model fitting included assessment for a delayed effect known as hysteresis and the justification for the choice of the concentration-QTc model. The term placebo-adjusted ΔQTcF in the concentration-QTc analysis was used to illustrate the underlying data on a subject level. It reflected the observed individual ΔQTcF on active treatment (lucerastat or moxifloxacin) or placebo minus the estimated mean ΔQTcF on placebo [20].

Assay sensitivity was evaluated by concentration-QTc analysis of the effect of 400 mg open-label moxifloxacin on ΔΔQTcF using a similar model as for the primary analysis. Assay sensitivity was deemed demonstrated if the slope of the moxifloxacin concentration-QTc relationship was statistically significant at the 10% level in a 2-sided test and the model-predicted QT effect was greater than 5 ms (i.e., the lower bound of the 2-sided 90% CI of ΔΔQTcF) at the observed geometric mean C_max_.

In addition, the effects of study treatment on ΔΔQTcF, ΔΔHR, ΔΔPR, and ΔΔQRS were evaluated at each post-dose time point (by-time point analysis) using the intersection union test. The by-time point analysis for QTcF was also based on a linear mixed-effects model with ΔQTcF as the dependent variable; period, sequence, time, study treatment, and time-by-treatment interaction as fixed effects; and baseline QTcF as a covariate. The same by-time point analysis model was used for HR, PR, and QRS as described for QTcF above. An unstructured covariance matrix was specified for the repeated measures at post-dose time points for subject within treatment period. From this analysis, the Least Squares (LS) mean and 2-sided 90% CI were calculated for the contrasts ‘lucerastat versus placebo’ at each dose of lucerastat and each post-dose time point, separately. The same was performed for moxifloxacin.

An analysis of categorical outliers was performed for changes in HR, PR, QRS, QTcF, T-wave morphology, and presence of U-waves. Categorical QTcF outliers were defined as QTcF values > 450 to ≤ 480, > 480 to ≤ 500, or > 500 ms and were analyzed descriptively, along with QTcF increases from baseline (defined as > 30 to ≤ 60 or > 60 ms).

Continuous demographic variables were summarized by descriptive statistics (mean ± SD), qualitative demographic characteristics were summarized by percentages, AEs and other safety parameters were summarized descriptively. In addition, PK parameters of lucerastat were summarized using geometric mean and two-sided 95% CI or median and range for t_max_.

All statistical analyses were performed using SAS® software (version 9.4 or higher; SAS Institute, Cary, NC, USA).

## Results

### Subject demographics and disposition

In Part A, 8 White male subjects were enrolled. All completed the study. The overall mean age was 31.8 years (range: 18–54 years) and the mean BMI was 23.4 kg/m^2^ (range: 19.9–28.1 kg/m^2^). There was no relevant imbalance in demographic characteristics between the subjects receiving lucerastat or placebo (data not shown). In Part B, 36 subjects were enrolled; 22 were females and 14 males. Of these, 24 were White, 9 Black or African American, 1 Asian, 1 Hispanic or Latino, and 1 mixed Aruban. The overall mean age was 29.6 years (range: 18–54 years) and the mean BMI was 23.9 kg/m^2^ (range: 19.3–29.4 kg/m^2^). One female subject was not dosed with open-label moxifloxacin in the 4th period due to difficulties in placing the cannula for blood withdrawals. Consequently, none of the scheduled assessments were performed. Therefore, data was available from 36 subjects on each dose of lucerastat and on placebo, and from 35 subjects on moxifloxacin.

### Pharmacokinetics

**Fig. 1:**
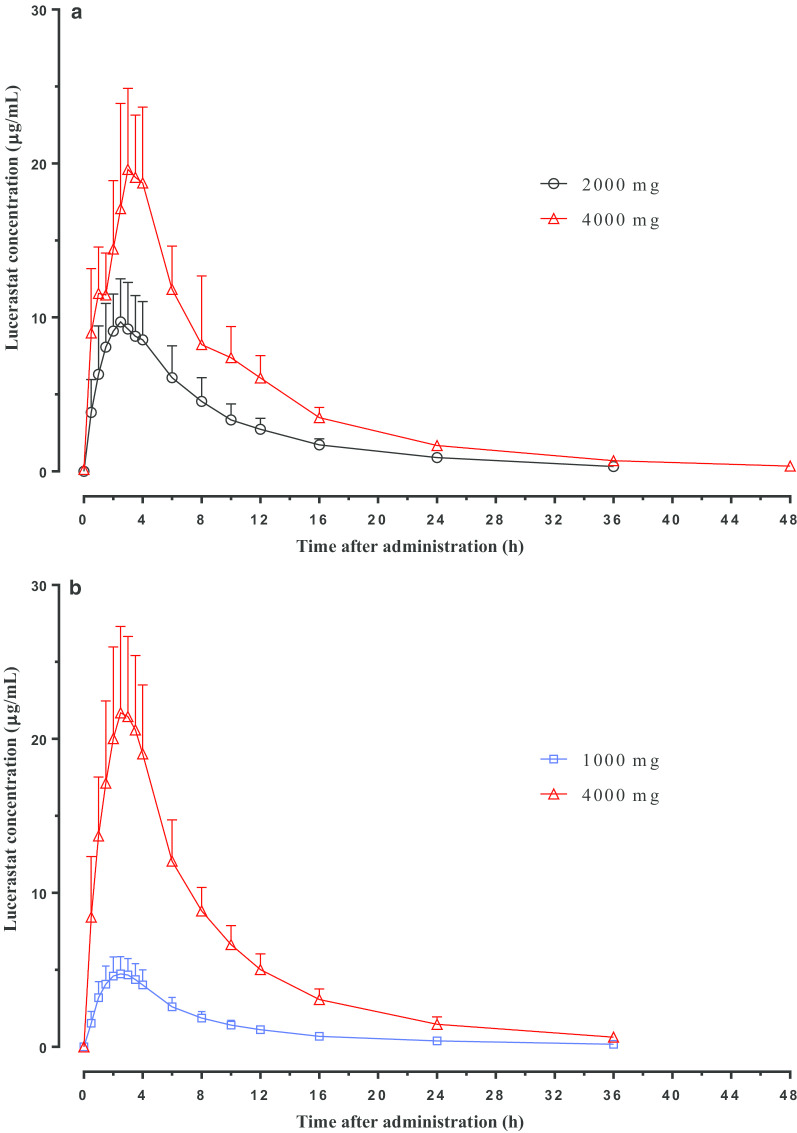
Plasma concentration–time profiles of lucerastat. Exposure to lucerastat increased approximately dose-proportionally and was characterized by rapid absorption and quick elimination. **a** Healthy male subjects after administration of 2000 and 4000 mg in Part A (N = 6). **b** Healthy male and female subjects after administration of 1000 and 4000 mg in Part B (N = 36). Data expressed as arithmetic mean (+ SD). N, number of subjects in the population; SD, standard deviation

A summary of lucerastat and moxifloxacin PK parameters is presented in Table 1. Across all doses, lucerastat was rapidly absorbed with peak plasma concentrations occurring between 0.5 and 4.0 h after dosing. Geometric mean t_½_ ranged from 8.0 to 10.0 h. The exposure to lucerastat increased dose-proportionally, i.e., was about 2- and 4.5-fold higher for C_max_ and AUC upon dosing with 4000 mg as compared to 2000 mg in Part A and 1000 mg in Part B, respectively. Mean plasma concentration–time profiles of lucerastat following single-dose administration in both parts are shown in Fig. 1. Based on PK and safety data, 4000 mg lucerastat was selected as the supratherapeutic dose to investigate the effect of lucerastat on cardiac repolarization in Part B. Peak plasma concentrations of moxifloxacin were reached at 2.0 h post-dose (median). Geometric mean C_max_ and AUC_0−t_ for moxifloxacin were 3.0 μg/mL and 32.6 μg⋅h/mL, respectively. Geometric mean t_½_ was 10.4 h [Table 1].

**Table 1:** Summary of lucerastat and moxifloxacin PK parameters

PK parameter (unit)	Lucerastat		
	2000 mg	4000 mg	
	n = 6	n = 6	
**Part A**			
C_max_ (µg/mL)	11.3 (9.9, 13.0)	22.1 (17.6, 27.7)	
t_max_ (h)	2.8 (1.5, 4.0)	3.3 (2.5, 4.0)	
AUC_0−t_ (µg·h/mL)	93.9 (75.7, 116.5)	193.7 (163.8, 229.1)	
AUC_0−∞_ (µg·h/mL)	98.1 (79.8, 120.5)	198.8 (167.7, 235.5)	
t_½_ (h)	8.0 (5.8, 11.0)	9.3 (7.1, 12.2)	

PK parameter (unit)	Lucerastat		Moxifloxacin
	1000 mg	4000 mg	400 mg
	n = 36	n = 36	n = 35
**Part B**			
C_max_ (µg/mL)	5.2 (4.9, 5.6)	24.3 (22.8, 26.0)	3.0 (2.7, 3.3)
t_max_ (h)	2.5 (1.5, 4.0)	2.5 (0.5, 4.0)	2.0 (0.5, 6.0)
AUC_0−t_ (µg·h/mL)	42.1 (39.8, 44.6)	191.1 (183.2, 199.2)	32.6 (30.1, 35.4)
AUC_0−∞_ (µg·h/mL)	45.0 (42.4, 47.7)	199.3 (190.9, 207.9)	35.9 (33.0, 39.2)
t_½_ (h)	10.0 (9.1, 11.0)	8.2 (7.6, 8.9)	10.4 (9.7, 11.1)

Data expressed as geometric mean (95% CI) or as median (range) for t_max_

AUC_0−∞_, area under the plasma drug concentration–time curve (from time zero to infinity); AUC_0−t_, area under the plasma drug concentration–time curve (from time zero to time t of the last measured concentration above the limit of quantification); CI, confidence interval; C_max_, maximum plasma concentration; n, number of subjects with available data; PK, pharmacokinetic; t_½_, half-life; t_max_, time to maximum plasma concentration

### Cardiodynamic ECG analysis

ECG parameters were well balanced across study treatments with mean HR between 61.5 and 63.5 bpm, QTcF between 403.0 and 406.5 ms, PR between 143.0 and 145.0 ms, and QRS between 102.5 and 102.8 ms.

**Fig. 2:**
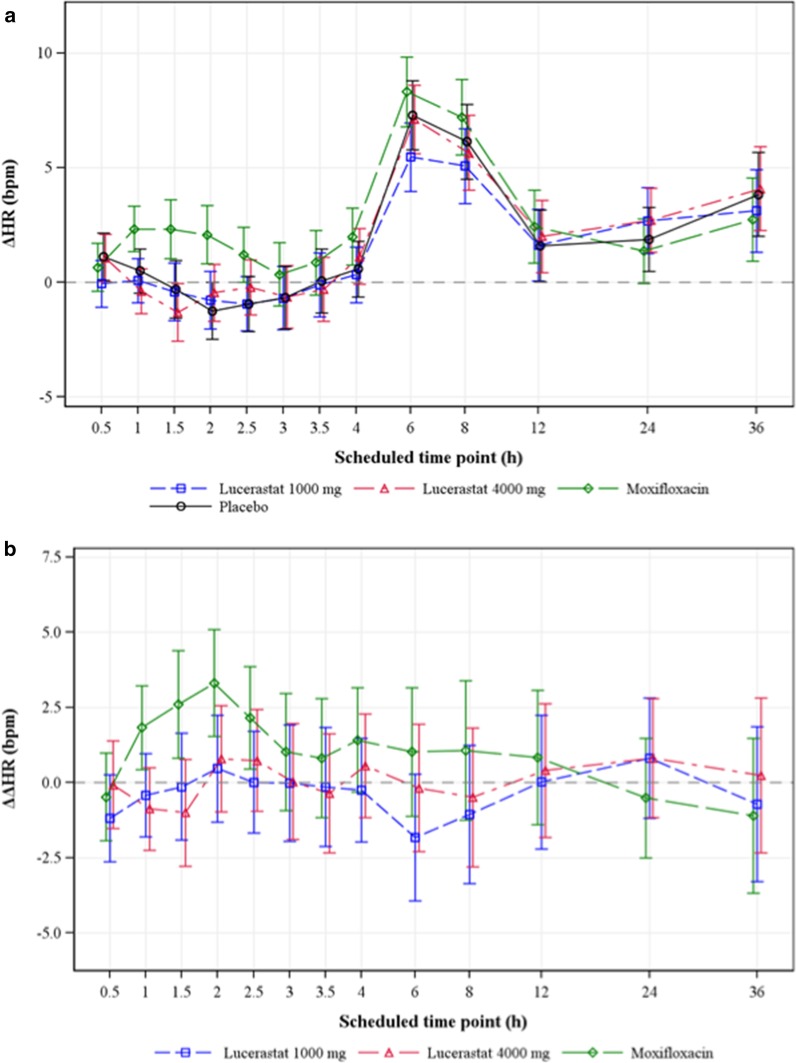
Change-from-baseline in HR across time points and study treatments. Mean ΔHR on lucerastat and moxifloxacin closely followed the pattern observed on placebo. **a** Effect of study treatments on ΔHR. **b** Effect of study treatments on ΔΔHR. Data expressed as LS mean and 90% CI based on a linear mixed-effects model. bpm, beats per minute; CI, confidence interval; Δ, change-from-baseline; ΔΔ, placebo-corrected change-from-baseline; HR, heart rate; LS, least squares

Lucerastat at single oral doses of 1000 and 4000 mg did not exert an effect on HR. The LS mean ΔHR on active treatment closely followed the pattern observed with placebo (Fig. 2a). Consequently, LS mean ΔΔHR varied within a narrow range across all post-dose time points following administration of lucerastat, i.e., between − 1.8 and 0.8 bpm (Fig. 2b).

**Fig. 3:**
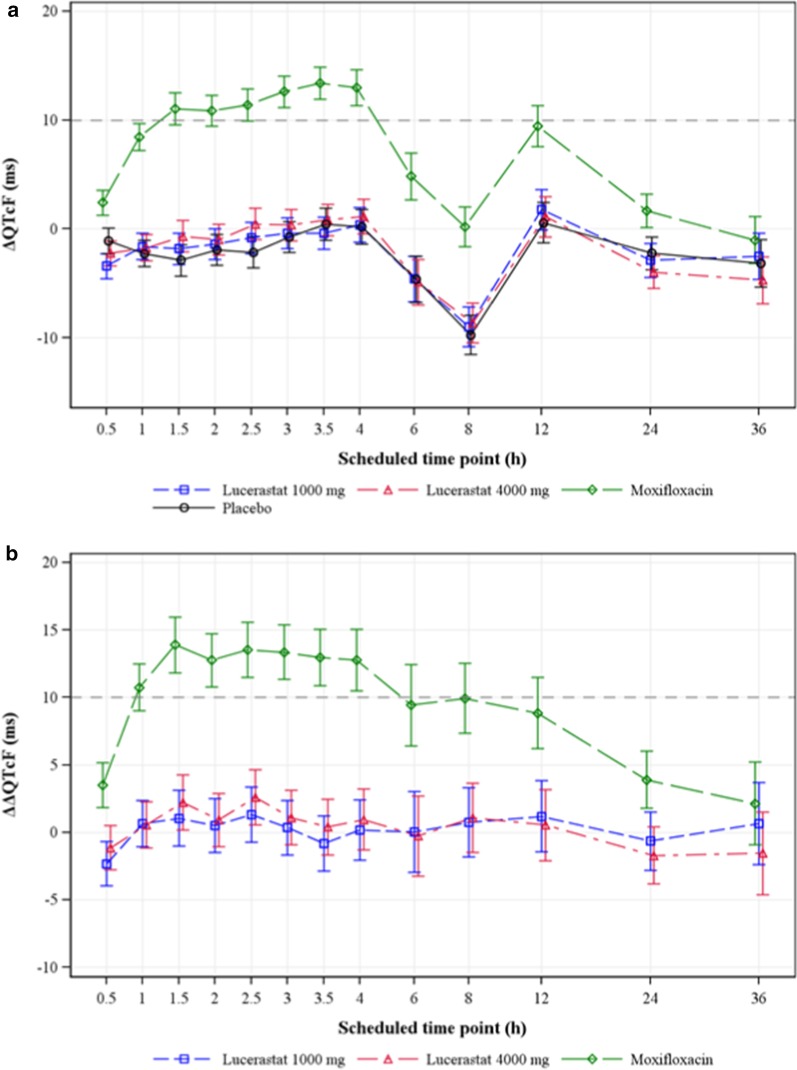
Change-from-baseline in QTcF across time points and study treatments. Mean ΔQTcF on lucerastat closely followed the pattern observed on placebo with no indication of dose dependency, whereas moxifloxacin caused a clear QTc prolongation. **a** Effect of study treatments on ΔQTcF. **b** Effect of study treatments on ΔΔQTcF. Data expressed as LS mean and 90% CI based on a linear mixed-effects model. The dashed black lines represent the threshold for clinically concerning QTc prolongation. CI, confidence interval; Δ, change-from-baseline; ΔΔ, placebo-corrected change-from-baseline; LS, least squares; QTcF, Fridericia-corrected QTc

By-time point analysis across all post-dose time points following administration of 1000 and 4000 mg lucerastat showed that the LS mean ∆QTcF on lucerastat closely followed the diurnal pattern observed on placebo with a reduction of ∆QTcF observed with all study treatments at 6 and 8 h post-dose (Fig. 3a). Consequently, the LS mean ∆∆QTcF on lucerastat varied between − 2.3 and 2.6 ms, without an indication of dose-dependency (Fig. 3b, Table 2). For both lucerastat doses, C_max_ and peak LS mean ΔΔQTcF occurred at 2.5 h post-dose, demonstrating the lack of hysteresis. In contrast to lucerastat, 400 mg oral moxifloxacin caused a clear QTc prolongation resulting in an increase of the LS mean ∆∆QTcF across post-dose time points with a peak value of 13.9 ms at 1.5 h post-dose and a lower bound of the 90% CI above 5 ms from 1 to 12 h post-dose (Fig. 3b, Table 2).

**Table 2:** ΔΔQTcF across study treatments and post-dose time points

Time post-dose (h)	Lucerastat		Moxifloxacin
	1000 mg	4000 mg	400 mg
	n = 36	n = 36	n = 35
0.5	− 2.3 (− 4.0, − 0.7)	− 1.1 (− 2.8, 0.5)	3.5 (1.9, 5.2)
1.0	0.7 (− 1.0, 2.4)	0.5 (− 1.2, 2.3)	10.7 (9.0, 12.5)
1.5	1.1 (− 1.0, 3.1)	2.2 (0.2, 4.3)	13.9 (11.8, 16.0)
2.0	0.5 (− 1.5, 2.5)	0.9 (− 1.0, 2.9)	12.8 (10.8, 14.8)
2.5	1.3 (− 0.7, 3.4)	2.6 (0.6, 4.7)	13.5 (11.5, 15.6)
3.0	0.4 (− 1.7, 2.4)	1.1 (− 0.9, 3.1)	13.4 (11.3, 15.4)
3.5	− 0.8 (− 2.9, 1.3)	0.4 (− 1.7, 2.5)	13.0 (10.9, 15.1)
4.0	0.2 (− 2.1, 2.4)	1.0 (− 1.3, 3.2)	12.8 (10.5, 15.1)
6.0	0.0 (− 3.0, 3.0)	− 0.3 (− 3.3, 2.7)	9.4 (6.4, 12.5)
8.0	0.7 (− 1.8, 3.3)	1.1 (− 1.5, 3.7)	9.9 (7.3, 12.5)
12.0	1.2 (− 1.4, 3.8)	0.6 (− 2.1, 3.2)	8.9 (6.2, 11.5)
24.0	− 0.6 (− 2.8, 1.5)	− 1.7 (− 3.8, 0.4)	3.9 (1.8, 6.0)
36.0	0.7 (− 2.4, 3.7)	− 1.5 (− 4.6, 1.5)	2.1 (− 0.9, 5.2)

Data expressed as LS means (90% CI) in ms

CI, confidence interval; Δ, change-from-baseline; ΔΔ, placebo-corrected change-from-baseline; ECG, electrocardiography; LS, least squares; n, number of subjects receiving lucerastat or moxifloxacin with available data; QTcF, Fridericia-corrected QTc

**Fig. 4:**
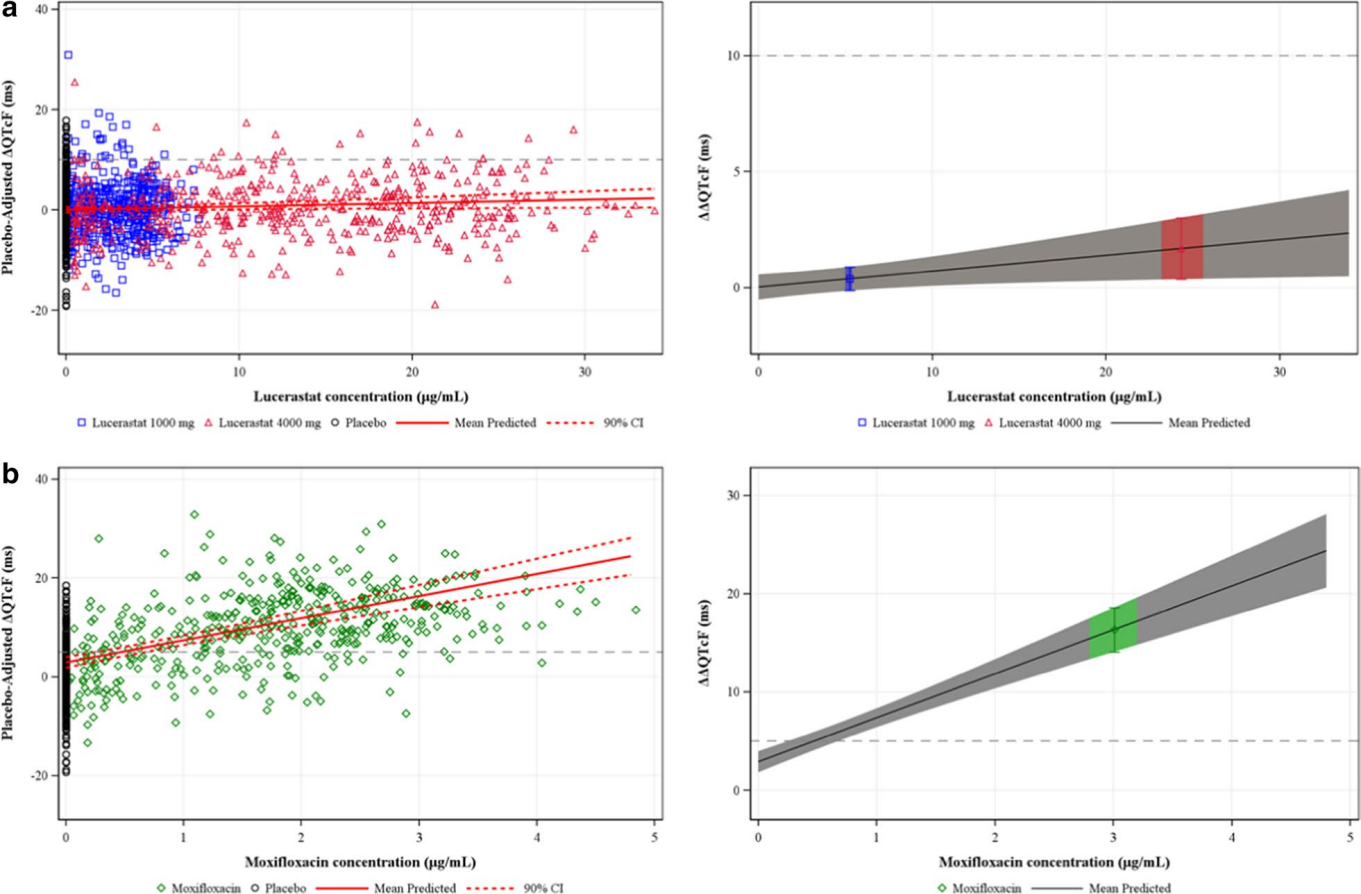
Concentration-QTc analysis. A linear model with a treatment effect-specific intercept was fitted for lucerastat and moxifloxacin plasma concentrations. For lucerastat, a QTcF effect above 10 ms could be excluded up to a plasma concentration of approximately 34 μg/mL, whereas moxifloxacin caused a clear QTc prolongation. **a** Lucerastat and **b** moxifloxacin. Left panel: scatter plots of observed plasma concentrations and estimated placebo-adjusted ΔQTcF by subject. The solid red lines with dashed red lines denote the predicted mean ΔΔQTcF with 90% CI. The blue squares, red triangles, and black circles denote the pairs of observed lucerastat plasma concentrations and estimated placebo-adjusted ΔQTcF by subject for lucerastat 1000 mg, lucerastat 4000 mg, and placebo, respectively (**a**). The green diamonds and black circles denote the pairs of observed moxifloxacin plasma concentrations and estimated placebo-adjusted ΔQTcF by subject for moxifloxacin and placebo, respectively (**b**). Right panel: relationship between lucerastat (**a**) and moxifloxacin (**b**) plasma concentrations and predicted ΔΔQTcF. The solid black lines with gray shaded area denote the predicted mean (90% CI) ΔΔQTcF. The blue, red, and green areas denote the predicted mean (90% CI) ΔΔQTcF with blue square, red triangle, and green diamond at the geometric mean (90% CI) C_max_ of lucerastat 1000 mg, lucerastat 4000 mg, and moxifloxacin 400 mg, respectively. The dashed black lines represent the thresholds for clinically concerning QTc prolongation (**a**) and for demonstrating assay sensitivity (**b**). CI, confidence interval; C_max_, maximum plasma concentration; Δ, change-from-baseline; ΔΔ, placebo-corrected change-from-baseline; QTcF, Fridericia-corrected QTc

In the concentration-QTc analysis, a linear model with a treatment effect-specific intercept was fitted for lucerastat plasma concentrations, which represented the data in an acceptable way. The relationship between the individually observed lucerastat plasma concentrations and estimated placebo-adjusted ΔQTcF is shown in Fig. 4a (left panel). As depicted in Fig. 4a (right panel) and Table 3, the estimated population slope of the lucerastat concentration-QTc relationship was shallow and statistically significant, i.e., 0.07 ms per μg/mL (90% CI 0.01–0.13; *p* = 0.0618) with a negligible, statistically nonsignificant treatment effect-specific intercept of 0.03 ms (90% CI − 0.51–0.57; *p* = 0.9307). Using this concentration-QTc relationship, the effect of lucerastat on ∆ΔQTcF was predicted to 0.39 ms (90% CI − 0.13 to 0.90) and 1.69 ms (90% CI 0.33–3.05) at the observed geometric mean lucerastat C_max_ upon dosing with 1000 mg (5.2 μg/mL) and 4000 mg (24.3 μg/mL), respectively. By looking at the upper bound of the 90% CI of the predicted effect (Fig. 4a, right panel), a QTcF effect above 10 ms could be excluded within the full range of lucerastat plasma concentrations, up to approximately 34 μg/mL.

**Table 3:** Summary of concentration-QTc analysis

Study treatment	Geometric mean C_max_ (90% CI) (μg/mL)	ΔΔQTcF Estimate (90% CI) (ms)	Concentration-QTc slope (90% CI) (ms per μg/mL)	Treatment effect-specific intercept (90% CI) (ms)
**Lucerastat**				
1000 mg	5.2 (4.96, 5.55)	0.39 (− 0.13, 0.90)	0.07 (0.01, 0.13) *p* = 0.0618	0.03 (− 0.51, 0.57) *p* = 0.9307
4000 mg	24. 3 (23.05, 25.68)	1.69 (0.33, 3.05)		
**Moxifloxacin**				
400 mg	3.0 (2.78, 3.25)	16.35 (14.03, 18.68)	4.47 (3.58, 5.36) *p* < 0.0001	2.89 (1.82, 3.97) *p* < 0.0001

CI, confidence interval; C_max_, maximum plasma concentration; Δ, change-from-baseline; ΔΔ, placebo-corrected change-from-baseline; QTcF, Fridericia-corrected QTc

The relationship between the individually observed moxifloxacin plasma concentrations and estimated placebo-adjusted ΔQTcF is shown in Fig. 4b (left panel). The estimated population slope of the moxifloxacin concentration-QTc relationship was 4.47 ms per μg/mL (90% CI 3.58–5.36); with a treatment effect-specific intercept of 2.89 ms (90% CI 1.82–3.97). Both the treatment effect-specific intercept and the slope of the relationship were statistically significant (*p* < 0.0001). The lower bound of the two-sided 90% CI of the predicted ∆ΔQTcF effect (16.35 ms [90% CI 14.03–18.68]) at the geometric mean moxifloxacin C_max_ (3.0 μg/mL) was above 5 ms (Fig. 4b right panel, Table 3).

Lucerastat at single oral doses of 1000 and 4000 mg did not have a clinically relevant effect on cardiac conduction, i.e., PR and QRS intervals (data not shown).

No outliers in terms of HR were observed except for 1 subject with HR below 50 bpm with a decrease-from-baseline > 25% at 1 time point after administration of 1000 mg lucerastat. A QTcF value between 450 and 480 ms was observed in 3 subjects (1 on lucerastat 4000 mg, 2 on moxifloxacin, and 1 on placebo). There were no subjects with QTcF > 480 ms or ΔQTcF > 30 ms. No treatment-emergent T-wave morphology changes were observed except for 3 observations of negative T-waves that occurred in the same subject at 1 time point each after administration of lucerastat 1000 mg, moxifloxacin, and placebo. No presence of U-waves was observed.

### Safety and tolerability

Lucerastat at single oral doses of 1000, 2000, and 4000 mg was safe and well tolerated. Moxifloxacin at a single oral dose of 400 mg was also safe and well tolerated. A summary of TEAEs reported in both parts is presented in Table 4. No serious AEs or any AEs leading to study treatment/study discontinuation were reported.

**Table 4:** Summary of treatment-emergent AEs by treatment

	Lucerastat						Placebo					
	2000 mg			4000 mg			Day 1			Day 3		
	Day 1			Day 3								
	N = 6			N = 6			N = 2			N = 2		
	nAEs	n	%	nAEs	n	%	nAEs	n	%	nAEs	n	%
**Part A**												
Any PT												
Number of subjects with at least one AE		1	16.7					1	50.0			
Number of different AEs	2						2					
Total number of AEs	2						2					
Most common AEs^a^												
Nausea	–	–	–	–	–	–	1	1	50.0	–	–	–
Vomiting	–	–	–	–	–	–	1	1	50.0	–	–	–
Fatigue	1	1	16.7	–	–	–	–	–	–	–	–	–
Nasal congestion	1	1	16.7	–	–	–	–	–	–	–	–	–

	Lucerastat						Moxifloxacin			Placebo		
	1000 mg			4000 mg			400 mg					
	N = 36			N = 36			N = 35			N = 36		
	nAEs	n	(%)	nAEs	n	(%)	nAEs	n	(%)	nAEs	n	(%)
**Part B**												
Any PT												
Number of subjects with at least one AE		12	33.3		9	25.0		18	51.4		9	25.0
Number of different AEs	9			13			12			9		
Total number of AEs	16			17			30			12		
Most common AEs^a^												
Headache	4	4	11.1	3	2	5.6	4	4	11.4	4	4	11.1
Dizziness	2	2	5.6	2	2	5.6	4	4	11.4	–	–	–
Nausea	–	–	–	–	–	–	6	6	17.1	–	–	–
Medical device site reaction	3	3	8.3	–	–	–	1	1	2.9	1	1	2.8
Electrocardiogram QT prolonged	–	–	–	1	1	2.8	8	3	8.6	–	–	–
Catheter site pain	–	–	–	2	2	5.6	1	1	2.9	–	–	–
Catheter site related reaction	2	2	5.6	1	1	2.8	–	–	–	–	–	–

^a^Occurring with a frequency ≥ 5% with any treatment

%, percentage of subjects based on N; AE, adverse event; N, number of subjects in the population; n, number of subjects with available data; PT, preferred term

On Day 1 in Part A, 1 subject on 2000 mg lucerastat and 1 subject on placebo reported each 2 TEAEs. No TEAE was reported following administration of 4000 mg lucerastat.

In Part B, 12 subjects on 1000 mg lucerastat, 9 subjects on 4000 mg lucerastat, 18 subjects on 400 mg moxifloxacin, and 9 subjects on placebo reported at least one TEAE. There were no clear differences in the incidence of TEAEs across doses of lucerastat or as compared to placebo. All TEAEs were graded by the investigator as mild in intensity and the most commonly reported TEAE was headache.

Overall, 9 treatment-emergent ECG abnormalities in 3 out of 36 subjects were judged by the investigator as clinically significant (8 after administration of 400 mg moxifloxacin, 1 after administration of 4000 mg lucerastat). These abnormalities were reported as TEAEs of ‘ECG QT prolonged’ as displayed in Table 4. Eight occurred following administration of 400 mg moxifloxacin in 3 out of 35 subjects (8.6%). One occurred following administration of 4000 mg lucerastat in 1 out of 36 subjects (2.8%). This subject showed a QTcF of 452 ms (+ 12 ms from baseline) at 2.5 h and again at 36 h after administration of 4000 mg lucerastat, where at the latter time point the investigator judged the excursion as not clinically significant. Considering that (a) the QTcF interval for this subject was only marginally above the upper limit of normal of 450 ms; (b) the change-from-baseline for QTcF was below 30 ms; (c) the increase occurred at the same magnitude also at 36 h post-dose, which was not considered clinically significant; and (d) this increase was not observed in the analysis of the continuous 24-h Holter ECG recording, these ECG findings were considered as not clinically relevant.

No clinically relevant changes in vital signs, body weight, or laboratory variables were identified upon administration of single oral doses of up to 4000 mg lucerastat.

## Discussion

This TQT study investigated the effect of the glucosylceramide synthase inhibitor lucerastat on cardiac repolarization. It was conducted as a prospective, single-center, randomized, double-blind (for lucerastat), placebo-controlled, two-part phase 1 study in healthy subjects. The selection of healthy subjects was justified on the basis that safety, tolerability, and PK can be investigated accurately in this population, without interference from concomitant diseases or medication. Part A was a pilot study in healthy male subjects to determine the supratherapeutic dose of lucerastat for Part B. Only male subjects were included in Part A to have a more homogeneous population and to avoid any potential variability in PK parameters due to menstrual cycle. Part B was a four-way crossover study in healthy male and female subjects including a therapeutic and supratherapeutic dose of lucerastat, open-label moxifloxacin as a positive, and placebo as a negative control. Using a healthy subject population is in line with regulatory guidance, which states that the TQT study is typically carried out in healthy subjects (as opposed to individuals at increased risk of arrhythmias) [18]. The intention of the TQT study in healthy subjects is to evaluate whether the drug causes clinically concerning QTc prolongation, not to directly assess the pro-arrhythmic potential in a clinical setting. Susceptible patients may develop pronounced QTc prolongation and torsades de pointes at drug concentrations that can be safely achieved in healthy subjects. To the best of our knowledge, examples of a drug causing QTc prolongation in patients only, but not in healthy subjects, do not exist, provided the drug has been evaluated in healthy subjects at sufficiently high exposure.

Both male and female subjects were included since females have a longer QTc interval and an increased risk of pro-arrhythmias caused by drug-induced delayed cardiac repolarization [25]. In addition, current guidance is encouraging, although not mandating, to include both sexes in a TQT study [19]. A crossover design with 4 periods separated by a washout period was chosen to allow intra-subject comparison of the 4 study treatments, thus, reducing variability and, therefore, decreasing the number of subjects needed for the study [26, 27]. Since moxifloxacin was given open-label, 3 4 × 4 Williams squares were used for randomization to retain the study blind and to ensure that first-order carry-over effects were balanced.

Regulatory guidance recommends that, if not precluded by considerations of safety or tolerability, a drug should be tested in TQT studies at substantial multiples of the anticipated maximum therapeutic exposure [18]. Since clinical experience with doses higher than the anticipated therapeutic dose of 1000 mg lucerastat twice daily was lacking, Part A investigated the safety, tolerability, and PK of 2000 and 4000 mg lucerastat. Based on results from Part A, the supratherapeutic dose of 4000 mg lucerastat was selected for Part B. This dose is regarded as a substantial multiple considering the following observations. Lucerastat is largely eliminated unchanged via renal excretion [15]. Therefore, it is not expected that hepatic impairment will affect the PK of lucerastat. In subjects with renal impairment, the exposure to lucerastat increased depending on the severity of their disease. Whereas the PK characteristics of lucerastat in subjects with mild renal impairment and healthy subjects were similar, the AUC_0−∞_ of lucerastat was 1.6- and 3.2-fold higher in subjects with moderate and severe renal impairment, respectively [28]. Therefore, dose reductions must be applied in subjects with moderate or severe renal impairment leading to similar exposure to lucerastat as in subjects with normal kidney function. Lucerastat is not prone to drug-drug-interactions and no such interaction is expected to increase exposure to lucerastat more than 1.2-fold. Results from a clinical drug-drug-interaction study with the organic cation transporter 2 inhibitor cimetidine in healthy male subjects demonstrated that cimetidine had only a weak effect on the exposure to lucerastat, i.e., led to an increase of geometric mean C_max_ and AUC by about 4 and 23%, respectively, at cimetidine steady state [29]. Food intake did not affect the total systemic exposure to lucerastat in a clinically relevant manner [15]. Taken together, there are no intrinsic or extrinsic factors expected to increase exposure to lucerastat combined to more than approximately 1.5-fold, provided the dose reductions for subjects with renal impairment are applied.

The use of single-dose administration to investigate the QTc effects of lucerastat is in line with current guidance that indicated that for drugs with a short t_½_ and no metabolites a single dose study might be sufficient [18], considering that lucerastat has indeed a short t_½_, does not accumulate, and is not metabolized to any great extent [15, 28].

Employing concentration-QTc analysis as primary analysis is in line with a recent revision of the applicable regulatory guidance [19] and the recently published white paper outlining the statistical principles [20]. Hence, it is considered appropriate to determine the effect of lucerastat at therapeutic and supratherapeutic doses on the QTc interval. Placebo is required in concentration-QTc analysis to correct for spontaneous diurnal variation, to control for potential bias introduced by study procedures, and to increase the power to exclude modest QTc effects in small-sized studies. The use of moxifloxacin as a positive control to detect small increases in QTc from baseline allowed appropriate assessment of assay sensitivity. The dose of 400 mg is the therapeutic dose and commonly used in TQT studies since it has been reproducibly shown to prolong the QTc interval [30, 31].

The moxifloxacin QTc response observed in this study clearly demonstrated assay sensitivity. The linear model seemed to slightly overestimate the QTc effect at high moxifloxacin plasma concentrations and a statistically significant treatment effect-specific intercept, i.e., at concentration zero, indicated that the model specification was not optimal. However, since assay sensitivity was also demonstrated in the by-time point analysis, with the lower bound of the 90% CI of the largest mean ∆∆QTcF above 5 ms, no further efforts were made to improve the concentration-QTc model for moxifloxacin by fitting additional nonlinear models. The observed mean C_max_ of moxifloxacin occurred at 2 h post-dose. A difference of 30 min was observed between the moxifloxacin mean C_max_ and the maximum LS mean ΔΔQTcF of 13.9 ms at 1.5 h post-dose. However, the effect of moxifloxacin on ΔΔQTcF is well characterized [31] and the small difference below 1 h did not warrant further exploration. Overall, the effect of moxifloxacin observed in this study was in concordance with previously described effects [30, 31].

Based on concentration-QTc analysis, a QTcF effect above 10 ms could be excluded up to lucerastat plasma concentrations of approximately 34 μg/mL. For both lucerastat doses, C_max_ and peak LS mean ∆∆QTcF occurred at 2.5 h post-dose demonstrating the lack of hysteresis. This was confirmed by graphical displays (data not shown) The effect of both doses of lucerastat on ΔΔQTcF was negligible and the largest mean effect, i.e., 2.6 ms, occurred after dosing with 4000 mg. The reduction of ∆QTcF observed with all study treatments at 6 and 8 h post-dose might be explained by the QTc shortening effect of a standardized meal, i.e., in this study the administration of lunch at 4 h post-dose, which may be correlated to the postprandial increase in cardiac output and the effect of C peptide and glucose on cardiac repolarization as described previously [32, 33].

At the geometric mean C_max_ of 24.3 μg/mL after administration of 4000 mg lucerastat the upper bound of the 90% CI of the predicted ∆∆QTcF was 3.05 ms. Considering this and since the exposure to lucerastat increases approximately dose-proportionally it is not expected that in a clinical worst-case scenario lucerastat plasma concentrations might be reached leading to a QTcF effect of clinical concern, provided the dose reduction scheme for patients with renal function impairment is followed.

Lucerastat at single oral doses up to 4000 mg was safe and well tolerated in both parts of the study. Overall, the safety profile observed in this study was in accordance with previous clinical experience.

In this TQT study, the anticipated therapeutic dose of 1000 mg and a supratherapeutic dose of 4000 mg lucerastat had no clinically relevant effect on the QTcF interval. This will be an important factor in the overall benefit-risk assessment of lucerastat in the potential treatment of FD since many Fabry patients suffer from cardiac morbidities including arrythmias.

## Conclusions

These results constitute a negative TQT study as described in the applicable regulatory guidance, demonstrating that lucerastat up to a single dose of 4000 mg does not have any clinically relevant liability to prolong the QT interval or any clinically relevant effect on other ECG parameters. In addition, lucerastat was safe and well tolerated even at the supratherapeutic dose, which represents a fourfold higher dose than the currently investigated therapeutic dose, covering a clinical worst-case scenario. The favorable safety profile of lucerastat observed in previous studies was confirmed.

## Data Availability

The datasets supporting the conclusions of this article are included within the clinical study report from the study presented in the manuscript. The key data supporting the findings of this study are available within the article.
